# Psychometric properties of the Chinese version of the oncology nurses health behaviors determinants scale: a cross-sectional study

**DOI:** 10.3389/fpubh.2024.1349514

**Published:** 2024-03-27

**Authors:** Yuxiu Liu, Lan Zhang, Shuzhen Li, Hua Li, Yuqi Huang

**Affiliations:** ^1^School of Nursing, Jinzhou Medical University, Jinzhou, China; ^2^Nursing Department, First Affiliated Hospital of Jinzhou Medical University, Jinzhou, China; ^3^Heze Home Economics College, Heze, China

**Keywords:** oncology nurses, chemotherapy exposure, health beliefs, factor analysis, psychometric evaluation

## Abstract

**Objective:**

To test the validity and reliability of the Oncology Nurses Health Behaviors Determinants Scale (HBDS-ON) in oncology nurses, the Chinese version was developed.

**Methods:**

The Brislin double translation-back translation approach was employed to forward translation, back translation, synthesis, cross-cultural adaptation, and pre-survey, resulting in the first Chinese version of the Oncology Nurses Health Behaviors Determinants Scale (HBDS-ON). A convenience sample technique was used to select 350 study participants in Liaoning, Shandong, and Jiangsu, China, who satisfied the inclusion and exclusion criteria, to assess the validity and reliability of the scale.

**Results:**

The Chinese version of the Oncology Nurses Health Behaviors Determinants Scale (HBDS-ON) had six subscales (perceived threat, perceived benefits, perceived barriers, self-efficacy, cues to action, and personal protective equipment availability and accessibility), including 29 items. The average scale level was 0.931, and the content validity level of the items varied from 0.857 to 1.000. Each Cronbach’s α coefficient had an acceptable internal consistency reliability range of 0.806 to 0.902. X^2/df^ = 1.667, RMSEA = 0.044, RMR = 0.018, CFI = 0.959, NFI = 0.905, TLI = 0.954, and IFI = 0.960 were the model fit outcomes in the validation factor analysis. All of the model fit markers fell within reasonable bounds.

**Conclusion:**

The Chinese version of the Oncology Nurses Health Behaviors Determinants Scale (HBDS-ON) has good reliability and validity and can be used as a tool to assess the influencing factors of chemotherapy exposure for oncology nurses in China.

## 1 Introduction

In recent years, the utilization of antineoplastic drugs has become increasingly prevalent in cancer treatment, mirroring the annual rise in cancer cases (1). However, the deleterious effects of these drugs on normal bodily functions have raised significant concerns, particularly among medical professionals who are regularly exposed to them (2). Among these professionals, oncology nurses stand out as a primary cohort frequently exposed to chemotherapy drugs, placing them at the highest risk of such exposures (3). Their exposure often occurs through various routes, including skin or mucosal contact, inadvertent ingestion, accidental needle pricks, or inhalation (4). Not only does this pose serious health risks to oncology nurses, consequently diminishing their quality of life, but it also directly impacts the quality of nursing care provided.

Adherence to safe chemotherapy-handling guidelines during chemotherapy procedures is crucial to prevent chemotherapy exposure (5). Research indicates that nurses often fail to fully comply with these guidelines when handling chemotherapy drugs (6). For instance, they may neglect to utilize essential personal protective equipment such as eye masks, chemo-specific gowns, and respiratory screens (4, 7).

Various factors hinder oncology nurses’ adherence to safe chemotherapy-handling guidelines, including their knowledge of these guidelines, workload, interpersonal influences, and support from workplace management (2). Interestingly, nurses’ health beliefs regarding chemotherapy exposures tend to have a greater impact on their adherence to the guidelines than their knowledge of such exposures (5).

The Health Belief Model (HBM) is a widely used framework for elucidating and predicting self-determined health behaviors aimed at preventing illnesses or other health issues. Within this model, health beliefs encompass perceived susceptibility to acquiring a disease, perceived severity of the condition, perceived benefits of engaging in health behaviors, perceived barriers to adopting such behaviors, and perceived self-efficacy in carrying out preventive health actions (8). Individuals’ preventive health actions are influenced by their health beliefs and cues to action (9). Moreover, the HBM illustrates how health attitudes are shaped by moderating factors such as age, gender, education, and expertise (10, 11).

Consequently, health beliefs regarding chemotherapy exposure and cues to adhere to protective measures serve as pivotal factors influencing oncology nurses’ compliance with chemotherapy-related occupational protective measures. By systematically evaluating these influential factors, we can establish a foundation for guiding nurses in adhering to chemotherapy-related occupational protective measures, thus facilitating the development of accurate intervention programs in the future.

The development of the Oncology Nurses Health Behaviors Determinants Scale (HBDS-ON) was grounded in the Health Belief Model (5). Accordingly, this study aims to translate the HBDS-ON into Chinese, assess the scale’s validity and reliability, and investigate its clinical utility among Chinese oncology nurses. This endeavor seeks to furnish a dependable and efficient tool for comprehensively assessing oncology nurses’ health beliefs concerning chemotherapy exposure, as well as the factors influencing adherence to guidelines and cues for adherence.

## 2 Materials and methods

### 2.1 Participants

In 2023, a cross-sectional study was conducted between March 1st and May 31th. Oncology nurses from Class Grade hospitals in Liaoning, Shandong, and Jiangsu provinces were selected as the study participants using convenience sampling. The inclusion criteria were as follows: (1) Nurses holding a valid nurse professional qualification certificate. (2) Willingness to participate voluntarily in the study. (3) Exposure to chemotherapy drugs for a duration of at least 1 year. Nurses were excluded if they: (1) Were absent from clinical duties due to reasons such as academic pursuits, vacation, pregnancy, or maternity leave during the survey period. (2) Did not engage in the allocation and administration of chemotherapy drugs before and after maternity leave and lactation. (3) Were involved in teaching or advanced studies within the surveyed hospitals.

To ensure the reliability of the analysis results, considering a sample size of 5 to 10 times the number of variables (12), a minimum of 200 cases were required for confirmatory factor analysis using a structural equation model (13). Accounting for a potential sample loss rate of 20%, 350 oncology nurses were targeted for inclusion in this study, and all participants provided informed consent before participation.

### 2.2 Translation and cultural adaptation of the HBDS-ON

This study utilized email communication to reach out to the original developers of the scale and secure authorization for translating the HBDS-ON scale into Chinese. The Brislin translation model was rigorously adhered to throughout the translation process (5).

#### 2.2.1 Forward translation

The original English version of the scale was translated into two Chinese versions, T1 and T2, by two native Chinese speakers (Care, Dr., and Nursing Postgraduate). The research team then convened to review and discuss the two Chinese versions of the questionnaire, addressing any contentious points and integrating them into the initial draft of the Chinese version, T3.

#### 2.2.2 Back translation

T3 was translated into English versions ET1 and ET2 by two native English teachers who had no prior exposure to the questionnaire. Subsequently, these translations were merged to create the English version ET3. The ET3 version was then submitted to the original author for feedback. Following a discussion and modification process involving members of the research team, the Chinese version of the scale, C1, was finalized.

#### 2.2.3 Cultural adaptation

The culturally adaptation of the Chinese version, C1, was carried out using the Delphi method. Seven experts, comprising three nursing management experts and four clinical oncology nursing experts, were recruited. All experts held associate senior titles and possessed bachelor’s degrees or higher (3 bachelor’s, 3 master’s, and 1 doctorate). The average years of professional experience among the experts were (25.57 ± 7.59) years, with all having more than 15 years of experience. Leveraging their clinical experience and professional expertise, the experts evaluated the context, cultural adaptability, and language expression of the items in the Chinese scale, C1, relative to the original scale. Subsequently, the research team incorporated expert feedback to modify the questionnaire, resulting in the formation of the revised Chinese version of the scale, C2.

### 2.3 Measurement and instruments

#### 2.3.1 General information questionnaire

The researchers created the general information questionnaire. Age, gender, educational background, professional experience in oncology, and technical title were among the data gathered.

#### 2.3.2 Chinese version of the HBDS-ON

The scale used to measure oncology nurses’ health behavior determinants that influence adherence to chemotherapy-handling guidelines was developed by American scholar Dania Abu-Alhaija in 2022 (5). It consists of 29 entries with 6 dimensions, including perceived threat, perceived benefits, perceived barriers, self-efficacy, cues to action, and personal protective equipment availability and accessibility. The Likert 5 scale scoring method was adopted: 1 = “strongly disagree,” 2 = “disagree,” 3 = “Neither agree nor disagree,” 4 = “agree,” and 5 = “strongly agree.” The HBDS-ON is a scale used to measure individual health beliefs and actions based on the Health Belief Model. The HBDS-ON is a scale measuring individual health beliefs and actions based on the Health Belief Model, consisting of a range of responses from “strongly disagree” to “strongly agree” and designed to assess the feasibility of implementing health behaviors (10, 14).

### 2.4 Data collection

#### 2.4.1 Pre-survey

In March 2023, 30 cases were selected from the cancer unit of a Grade-Three hospital in Liaoning Province. Informed consent was obtained from all participants, and the investigator provided them with an explanation of the study’s objectives and relevance. Participants were then asked to rate the acceptability of each item in terms of language, meaning, and content. All clinical oncology nurses reported that the items on the scale were easy to understand and had a clear format. Consequently, no changes were made to the questions in the Chinese version of the HBDS-ON for Oncology Nurses.

#### 2.4.2 Formal investigation

The Questionnaire Star platform was utilized for the survey. Prior to administering the survey, informed consent was obtained from the nursing department of each hospital. The head nurse of the oncology department was also contacted to communicate the research purpose and instructions for completing the questionnaire. Subsequently, the questionnaire was distributed to the WeChat group of each department.

At the onset of the questionnaire, guidance language was provided to clarify that the survey was solely for scientific research purposes. Additionally, participants were assured of the anonymity and voluntary nature of data collection. Participants completed the questionnaire online, and submission was only possible after all questions were answered.

Two weeks later, a subset of 30 oncology nurses randomly selected from the initial participants were re-surveyed using the same questionnaire to analyze test–retest reliability.

### 2.5 Data analysis method

SPSS 25.0 and AMOS 23.0 statistical software were used to analyze the data.

#### 2.5.1 Project analysis method

The critical ratio method and correlation coefficient method were used to screen the items on the scale. (1) Critical ratio method: 350 questionnaires were sorted according to the total score from high to low, and an independent sample t-test was used to compare whether the difference between the high group (the first 27%) and the low group (the last 27%) was statistically significant, and statistically significant items will be retained (15). (2) Correlation coefficient method: The Pearson correlation coefficient method was used to calculate the correlation coefficient between 29 items and the total amount table, and the items with a very weak correlation (*r* < 0.3) with the total score of the scale were deleted (16).

#### 2.5.2 Validity test method

(1) Content validity: Each item on the Chinese HBDS-ON scale was scored by seven experts, with 1 = irrelevant, 2 = weakly correlated, 3 = strongly correlated, and 4 = very correlated. Based on the expert evaluation results, the Item Level Content Validity Index (I-CVI) and the Scale Level Content Validity Index/Average (S-CVI/Ave) were computed. Generally, I-CVI ≥ 0.78 and CVI/Ave ≥ 0.9 (17). (2) Construct validity: Confirmatory Factor Analysis (CFA) was employed to assess the scale’s structural validity. The model fitting indexes mainly included Chi-square freedom ratio (χ^2^/df), Root-Mean-Square Error of Approximation (RMSEA), Root Mean Square Residual (RMR), Comparative Fit Index (CFI), Norm Fit Index (NFI), Tuck-Lewis Index (TLI), and Value added Fit Index (IFI). It is generally believed that χ^2^/df < 3, RMSEA<0.08, RMR < 0.05, CFI, NFI, TLI, IFI > 0.9 meet the model fitting requirements (18). (3) Convergent and discriminant validity: Average Variance Extracted (AVE), Combined Reliability (CR), and correlation coefficients between observed variables were calculated based on the results of CFA, and when AVE > 0.50, CR > 0.70,convergent validity was in the acceptable range (10). The discriminant validity was standardized when the correlation coefficients of all dimensions of the scale were less than the square root of AVE (10).

#### 2.5.3 Reliability test method

Reliability is an indicator of the accuracy or consistency of a measuring instrument’s response to a measurement and reflects the degree of truth of the measured characteristic (19). In this study, internal consistency and retest reliability were used for reliability testing. Cronbach’s α coefficient were calculated for the Chinese version of the scale and each dimension to evaluate the internal consistency of the scale. Thirty oncology nurses were selected according to the inclusion and exclusion criteria, and the Chinese version of the scale was used for the test–retest reliability, with a 2-week interval between the 2 measurements. The Intraclass Correlation Coefficient (ICC) was used to test the retest reliability of the two measurement scores with the aim of measuring the stability and consistency of the scale over time. The split-half reliability was then obtained by dividing the scale items into two halves and calculating the correlation between the results of each half.

#### 2.5.4 Ethical consideration

The Research Ethics Review Board of Jinzhou Medical University First Affiliated Hospital (JZMULL2023046) granted ethical approval for this study. Prior to commencing the survey, participants were briefed on the study’s objectives, the content of the survey, its voluntary nature, and the confidentiality of their personal information. Consent was obtained by having participants complete the survey voluntarily.

## 3 Results

### 3.1 Cross-cultural adaptation results

Based on the actual situation and language conventions in China, the items in the initial draft of the Chinese version were thoroughly reviewed and adjusted from the perspectives of semantics, idiomatic expressions, experience, and concepts. Following the feedback from experts, the research team implemented the following modifications: (1) In items 5, 8, 13, 15, 18, 19, 27, 28, and 29, the phrase “when performing chemotherapy operations” was revised to “when performing chemotherapy operations (including preparation, administration, handling of medical waste after medication, management of patient secretions and excretions, etc.).” This modification was made to facilitate nurses’ comprehension and prevent ambiguity, given that handling medications, such as preparation, patient administration, and disposal, typically involves exposure to chemotherapy drugs (20). (2) The statement “Personal protective equipment in our work area is stored in an inaccessible location” was amended to “Personal protective equipment in our work area is stored in a difficult-to-access location.”

### 3.2 Sample characteristics

There were 350 subjects in this survey. The basic information of the subjects, such as gender, age, working experience in oncology, education, and technical title, is displayed in Table 1 for more details on the sample’s demographic features.

**Table 1:** Oncology nurse participants’ demographics (*N* = 350).

Demographic variables	Categories	N	%
Gender	Female	345	98.6	Male	5	1.4
Ages (years)	20~<30	125	35.7	30~<40	177	50.6	40~<50	33	9.4	≥50	15	4.3
Oncology experience (years)	1 year to <5 years	158	45.1	5 year to <10 years	106	30.3	10 year to<15 years	57	16.3	15 year to<20 years	20	5.7	≥20 years	9	2.6
Education	Secondary	1	0.3	College	40	11.4	Undergraduate	288	82.3	Postgraduate	21	6
Technical title	Nurse	35	10	Nurse practitioner	180	51.4	Nurse-in-charge	125	35.7	Vice professor of nursing	9	2.6	Professor of nursing	1	0.3

### 3.3 Item analysis

The critical ratios (CR) of the 29 items of the scale in this study ranged from 7.834 to 11.876, all of which were greater than 3.000. *p* < 0.001 indicates that the items were well differentiated. Every item on the scale had a positive correlation with the overall score (r) (0.495 ~ 0.670), all >0.4, and *p* < 0.001, indicating a moderate correlation between each item and the scale. Eliminating item by item, the Chinese scale’s Cronbach’s α coefficient varied between 0.930 and 0.932, which does not exceed Cronbach’s α value of the scale (0.933). In conclusion, each item of the scale was retained (Table 2).

**Table 2:** Item analysis for the Chinese version of the HBDS-ON.

Item	Critical ratio	Correlation coefficient between item and total score	Cronbach’s alpha if the item delete
PT-1	8.434	0.576	0.931
PT-2	10.225	0.587	0.932
PT-3	8.971	0.608	0.931
PT-4	10.188	0.609	0.931
PT-5	10.226	0.593	0.931
PBe-1	8.53	0.522	0.931
PBe-2	8.132	0.524	0.931
PBe-3	9.539	0.543	0.932
PBa-1	10.297	0.606	0.932
PBa-2	10.899	0.612	0.931
PBa-3	9.276	0.613	0.931
PBa-4	10.392	0.637	0.931
PBa-5	10.765	0.605	0.931
SE-1	9.899	0.579	0.930
SE-2	10.033	0.593	0.931
SE-3	9.205	0.546	0.931
SE-4	9.932	0.565	0.931
SE-5	11.284	0.615	0.931
SE-6	9.957	0.604	0.931
CA-1	10.652	0.619	0.931
CA-2	10.021	0.577	0.931
CA-3	10.287	0.631	0.931
CA-4	11.565	0.670	0.931
CA-5	11.546	0.621	0.930
CA-6	11.876	0.634	0.930
CA-7	11.031	0.616	0.930
CA-8	11.159	0.628	0.930
PPE-1	10.214	0.586	0.931
PPE-2	7.834	0.495	0.930

PT, perceived threat; PBe, perceived benefits; PBa, perceived barriers; SE, self-efficacy; CA, cues to action; PPE, personal protective equipment availability and accessibility.

### 3.4 Validity

#### 3.4.1 Content validity

Seven experts evaluated the correlation between items and measured content through a 4-level scoring method (1 to 4 corresponding to “irrelevant” to “very relevant”). The content validity index S-CVI/Ave of the Chinese version of HBDS-ON was 0.931, and the item content validity index I-CVI was 0.857–1.000.

#### 3.4.2 Construct validity

**Figure 1:**
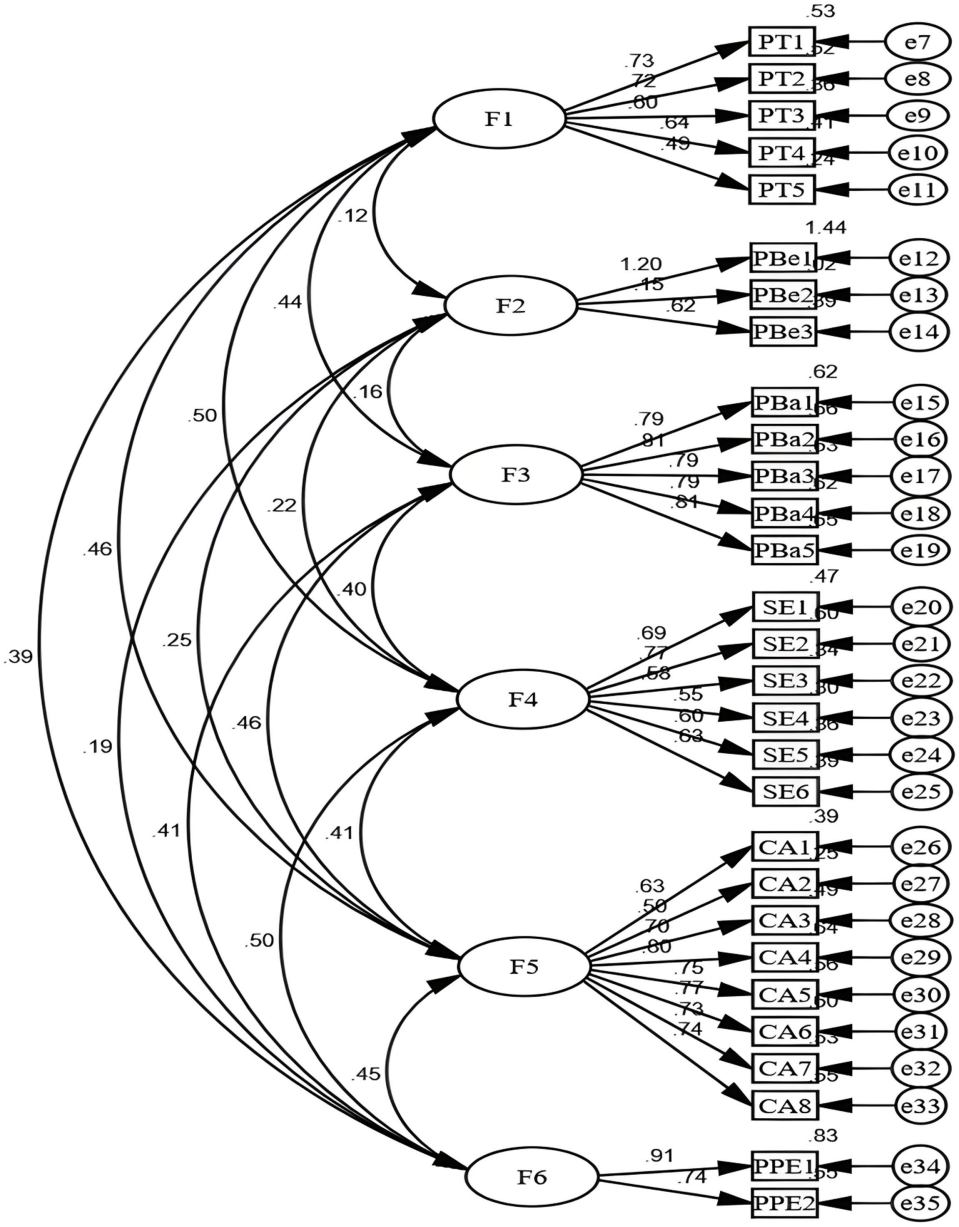
Standardized six-factor structural model ofthe Chinese HBDS-ON scale (*N* = 350). PT: Perceived Threat, PBe: Perceived Benefits, PBa: Perceived Barriers, SE: Self-Efficacy, CA: Cues to Action, PPE: Personal Protective Equipment availability and accessibility.

The survey data underwent confirmatory factor analysis (CFA) using Amos 23.0 software to construct the model. Subsequently, a structural equation model was derived (Figure 1). The goodness-of-fit index is presented in Table 3.

**Table 3:** The Chinese version of the HBDS-ON scale model fit index.

Item	CMIN/DF	RMSEA	RMR	CFI	NFI	TLI	IFI
Reference value	<3.000	<0.080	<0.050	>0.900	>0.900	>0.900	>0.900
model	1.667	0.044	0.018	0.959	0.905	0.954	0.960

According to the calculation formula, the combined reliability (CR) values of the six dimensions of the Chinese HBDS-ON scale were found to be 0.903, 0.823, 0.900, 0.894, 0.916, and 0.816, respectively, all exceeding the threshold of 0.80. Additionally, the average variance extracted (AVE) values were 0.651, 0.607, 0.644, 0.586, 0.575, and 0.692 for each dimension, respectively, all surpassing the criterion of 0.50. These results indicate that the convergent validity met the established criteria as outlined in Table 4. Furthermore, the square roots of the AVE values were found to be greater than the corresponding correlation coefficients, and the discriminant validity was within acceptable limits, as presented in Table 5. In summary, based on the above findings, it can be concluded that the Chinese version of the HBDS-ON scale demonstrates good structural validity.

**Table 4:** Convergent validity of the Chinese HBDS-ON scale.

Subscale	Item	Unstandardized factor loading	Standardized factor loading	CR	AVE
Perceived threat	PT-1	1.000	0.769	0.903	0.651	PT-2	1.047	0.793			PT-3	0.980	0.791			PT-4	1.094	0.865			PT-5	0.940	0.812		
Perceived benefits	PBe-1	1.000	0.802	0.823	0.607	PBe-2	0.910	0.732			PBe-3	1.096	0.802		
Perceived barriers	PBa-1	1.000	0.791	0.900	0.644	PBa-2	0.947	0.818			PBa-3	0.915	0.801			PBa-4	0.873	0.790			PBa-5	0.956	0.811		
Self-efficacy	SE-1	1.000	0.725	0.894	0.586	SE-2	1.490	0.820			SE-3	1.050	0.690			SE-4	1.058	0.766			SE-5	1.425	0.798			SE-6	1.156	0.786		
Cues to action	CA-1	1.000	0.753	0.916	0.575	CA-2	0.960	0.723			CA-3	1.172	0.793			CA-4	1.701	0.802			CA-5	0.990	0.745			CA-6	1.027	0.765			CA-7	1.029	0.741			CA-8	1.160	0.743		
Personal protective Equipment availability and accessibility	PPE-1	1.000	0.915	0.816	0.692	PPE-2	0.770	0.739			

**Table 5:** Discriminant validity of the Chinese HBDS-ON scale.

Subscale	PPE availability and accessibility	Cues to action	Self-efficacy	Perceived barriers	Perceived benefit	Perceived threat
PPE availability and accessibility	0.832	–	–	–	–	–
Cues to action	0.435	0.759	–	–	–	–
Self-efficacy	0.497	0.435	0.765	–	–	–
Perceived barriers	0.398	0.456	0.442	0.802	–	–
Perceived benefits	0.450	0.497	0.441	0.426	0.779	–
Perceived threat	0.402	0.452	0.496	0.425	0.394	0.807

The diagonal blue numbers are AVE square root values. PPE, personal protective equipment.

### 3.5 Reliability

The Chinese HBDS-ON scale’s Cronbach’s α coefficient was 0.933, whereas each subscale’s Cronbach’s α coefficient varied from 0.806 to 0.902, which was higher than English version (Cronbach alpha between 0.70 and 0.88), while the stability of the translated scale was found to be at a very favorable level (5). In addition, after 2 weeks, the test–retest reliability (ICC) was found to be 0.860.The split-half reliability was 0.796.

## 4 Discussion

In recent years, the increased utilization of antineoplastic drugs has led to a heightened risk of chemotherapy exposure among oncology nurses, resulting in continuous harm to their bodies (21, 22). Therefore, establishing a safe working environment to ensure the occupational protection of oncology nurses during chemotherapy is of utmost importance.

Currently, in China, the management of occupational safety protection related to antineoplastic drugs is still in its nascent stage, with no established guidelines or standards. However, hospitals conduct training sessions for oncology nurses on chemotherapy occupational protection knowledge, attitudes, and practices, and formulate related occupational protection measures. Studies have shown that the level of knowledge, attitudes, and behaviors regarding chemotherapy occupational protection among nursing staff in China is generally low to moderate, indicating a need for improvement (23). Additionally, there persists a misconception among some nursing staff that wearing protective gear is only necessary during the process of preparing chemotherapy drugs (24), leading to an increased risk of chemotherapy exposure for oncology nurses.

Utilizing a scientifically validated instrument to assess the factors influencing oncology nurses’ compliance with chemotherapy-related occupational safeguards is essential. Hence, the introduction of the HBDS-ON scale, which measures variables based on the Health Belief Model that influence oncology nurses’ adherence to chemotherapy handling guidelines.

The HBDS-ON scale assesses six areas: perceived threat, perceived benefits, perceived barriers, self-efficacy, cues to action, and availability and accessibility of personal protective equipment. It provides a valid and comprehensive description of the factors associated with oncology nurses’ compliance with chemotherapy-related occupational safeguards (25).

The Chinese version of the HBDS-ON scale was developed in this study by adhering to Brislin’s traditional translation paradigm, including forward translation, back translation, synthesis, cross-cultural adaptation, and pre-survey. According to item analysis, the Chinese version of the HBDS-ON demonstrated high item discrimination, with each item exhibiting a strong correlation with the scale. Even after the removal of each item, the Cronbach’s α coefficient remained consistent with the original value of the Chinese version of the scale (0.933). These findings suggest that all 29 items of the Chinese version of the HBDS-ON can be retained with good discrimination.

In this study, both the structural validity and content validity of the HBDS-ON scale’s Chinese version were thoroughly examined and assessed. Structural validity evaluates how well the theoretical assumptions of the scale align with the actual measurements, while content validity assesses whether the items adequately meet the needs and objectives of the measurement. The Chinese version of the HBDS-ON scale demonstrated good content validity, with item level content validity index (I-CVI) scores ranging from 0.857 to 1, and the scale level content validity index/average (S-CVI/Ave) of 0.931, surpassing the content validity reference value (26). These results indicate that the content evaluated by the scale is highly esteemed by professionals, and the scale’s straightforward language makes it suitable for use by oncology nurses.

Structural validity was examined using confirmatory factor analysis (CFA) for the Chinese version of the HBDS-ON scale. The results indicated a well-fitting structural equation model. Convergent validity, which assesses whether items measuring the same underlying constructs are grouped together, was confirmed as the CR values exceeded 0.8 and the AVE values exceeded 0.5 for all six subscales, indicating convergence (27). Discriminant validity, which tests whether items measuring different constructs are not grouped together, was confirmed as the square root of the AVE was greater than the correlation coefficient of each specific subscale, indicating good differentiation among the subscales (28).

The reliability of the Chinese version of the HBDS-ON was assessed using the test–retest (ICC), split-half, and internal consistency reliability methods. The Cronbach’s α coefficient greater than 0.8 is considered excellent, between 0.6 and 0.8 is good, and less than 0.6 is poor (29). The translated scale demonstrated highly favorable stability and the Cronbach’s α coefficient of 0.933, which is higher than the results of the English version. For the subscales, the Cronbach’s α coefficient ranged from 0.806 to 0.902. The split-half value was 0.796. Test–retest reliability is considered good if the ICC is >0.75, better if the ICC is ≥0.4 and ≤ 0.75, and poor if the ICC is <0.4 (29). The ICC value measured after 2 weeks was 0.860, indicating that the Chinese version of the scale has good retest reliability. Therefore, the scale can be used to assess factors affecting oncology nurses’ adherence to chemotherapy practice guidelines for chemotherapy safety with good temporal stability.

In conclusion, the Chinese version of the HBDS-ON scale exhibits strong structural validity, content validity, and reliability, making it a reliable tool for assessing factors influencing oncology nurses’ adherence to chemotherapy handling guidelines for safe chemotherapy treatment.

## 5 Limitations

The present study has several limitations that need to be acknowledged. Firstly, although the sample size met the criteria for the study, the study was conducted in only three regions and may not be fully representative of oncology nurses across the country. This limitation may hinder the broad applicability and generalizability of the scale. In addition, principal component analysis was not used in this study to determine whether the structure of the original questionnaire was retained. Future studies should conduct further validation and include multi-center large sample studies to explore the applicability of the scale in different regions.

## 6 Conclusion

Additionally, while this study effectively introduced the English version of the Oncology Nurses Health Behaviors Determinants Scale (HBDS-ON) to China and followed the Brislin translation process meticulously, there may still be some cultural nuances and linguistic differences that were not fully addressed. Further validation and adaptation of the scale may be necessary to ensure its relevance and appropriateness within the Chinese context. Despite these limitations, this study provides a reliable instrument for evaluating the factors influencing oncology nurses’ adherence to occupational safety precautions related to chemotherapy. By doing so, it contributes to the provision of excellent care for patients by ensuring the health and safety of oncology nurses and lays the groundwork for future research endeavors aimed at developing interventions and training materials in this critical area.

## Data availability statement

The raw data supporting the conclusions of this article will be made available by the authors, without undue reservation.

## Ethics statement

The studies involving humans were approved by Jinzhou Medical University First Affiliated Hospital’s Research Ethics Review Board. The studies were conducted in accordance with the local legislation and institutional requirements. The participants provided their written informed consent to participate in this study.

## Author contributions

YL: Conceptualization, Data curation, Formal analysis, Investigation, Methodology, Software, Validation, Writing – original draft, Writing – review & editing. LZ: Funding acquisition, Methodology, Project administration, Resources, Supervision, Visualization, Writing – review & editing. SL: Investigation, Methodology, Resources, Writing – review & editing. HL: Investigation, Methodology, Software, Writing – review & editing. YH: Data curation, Formal analysis, Investigation, Writing – review & editing.
